# Frailty assessment in kidney transplantation: insights from a European survey

**DOI:** 10.1093/ckj/sfag065

**Published:** 2026-02-26

**Authors:** Arzu Velioglu, Helen Erlandsson, Erol Demir, Rachel Hellemans, Ilaria Gandolfini, Annelis de Weerd, Ivana Dedinska, Adnan Sharif, Mario Schiffer, Luuk Hilbrands, Christophe Mariat

**Affiliations:** Department of Nephrology, Marmara University School of Medicine, Istanbul, Türkiye; Department of Clinical Science, Intervention and Technology, Division of Renal Medicine, Karolinska Institutet, Stockholm, Sweden; Transplant Immunology Research Centre of Excellence, Koç University Hospital, Istanbul, Türkiye; Department of Internal Medicine, Division of Nephrology, Yeditepe University School of Medicine, Istanbul, Türkiye; Department of Nephrology-Hypertension, Antwerp University Hospital, Edegem, Belgium; Faculty of Medicine & Health Sciences, Laboratory of Experimental Medicine and Paediatrics (LEMP), University of Antwerp, Wilrijk, Belgium; Nephrology Unit, University Hospital of Parma, Parma, Italy; Department of Internal Medicine, Erasmus MC Transplant Institute, University Medical Center Rotterdam, Rotterdam, The Netherlands; Transplant-Nephrology Department, Jessenius Faculty of Medicine, Comenius University and University Hospital Martin, Martin, Slovakia; Department of Nephrology and Transplantation, University Hospitals Birmingham, Queen Elizabeth Hospital, Birmingham, UK; Department of Nephrology and Hypertension, University Hospital Erlangen, Friedrich-Alexander University Erlangen-Nürnberg, Erlangen, Germany; Department of Nephrology, Radboud University Medical Center, Nijmegen, The Netherlands; Service de Néphrologie, Dialyse et Transplantation Rénale, Hôpital Nord, CHU de Saint-Etienne, Université Jean Monnet, Saint-Etienne, France

**Keywords:** frailty, kidney transplantation, outcome, screening, survey

## Abstract

**Background:**

Frailty has been identified as an important determinant of adverse transplant-related outcomes; however, its integration into routine kidney transplant practice remains limited.

**Methods:**

This was a web-based cross-sectional survey assessing current practice patterns and perceptions on frailty assessments among transplant physicians distributed by the DESCaRTES working group of the European Renal Association. The survey consisted of questions about frailty perceptions and practices both pre- and post-transplant.

**Results:**

A total of 134 responses (response rate 39.2%) were recorded. Although 98.5% recognized frailty as a risk factor for adverse outcomes, only 6.7% reported routinely performing a standardized frailty assessment in pre-transplant evaluations. Nearly 30% never evaluated frailty, and 24.6% did so only in selected high-risk patients, primarily based on age, comorbidities or prolonged dialysis duration. Physical function was recognized as the most important component of frailty assessments; however, no single assessment tool was consistently implemented. The main barriers to integrating frailty assessments into clinical practice were lack of trained staff, absence of standardized guidelines, and uncertainty about clinical usefulness. Post-transplant frailty assessments were rarely conducted routinely, despite 74.6% acknowledging their potential value.

**Conclusions:**

Despite widespread recognition of frailty as a significant determinant influencing transplant outcomes, its routine assessment in clinical practice remains limited across European centers. Variability in assessment tools, lack of guidelines and insufficient training resources contribute to this gap. Addressing these barriers through targeted education, validation of practical assessment tools, and consensus-driven protocols is essential to support the integration of frailty evaluation into both pre- and post-transplant care.

## GRAPHICAL ABSTRACT

**GRAPHICAL ABSTRACT:**
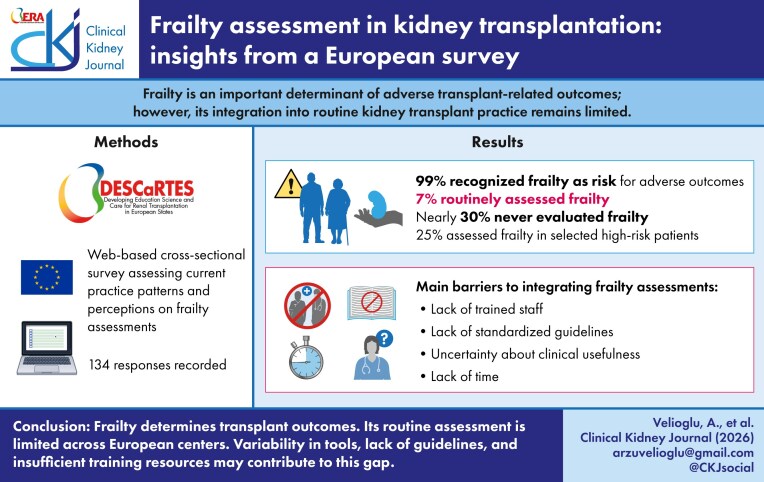

KEY LEARNING POINTS **What was known:**

- Frailty is associated with an increased risk of adverse outcomes in kidney transplantation, including delayed graft function, longer hospital stays and mortality.
- The prevalence of frailty has been reported to be 17%–21% among waitlisted patients and ∼15%–20% among kidney transplant recipients.
- Despite growing awareness, frailty assessments in kidney transplantation practice remain inconsistently performed.

**This study adds:**

- To the best of our knowledge, this is the first European-wide survey to explore how transplant physicians perceive frailty and how (or whether) they incorporate frailty assessment into clinical practice.
- While most clinicians recognize frailty as an important risk factor, only a minority of centers use standardized tools routinely.
- There is considerable variability in the tools used; perceived barriers to implementation in clinical practice are insufficient trained staff and lack of guidelines.

**Potential impact:**

- Our findings highlight a clear gap between the recognition of frailty’s clinical importance, and its routine use in practice.
- These insights may promote development of consensus guidelines, training programs and integrated care aimed at identifying and optimally managing frail kidney transplant candidates and recipients.

## INTRODUCTION

Frailty is a clinical condition characterized by decreased functional reserve, increased susceptibility to stressors, and increased risk of unfavorable outcomes [1]. It is now considered a major risk factor for adverse outcomes among patients of all ages with end-stage kidney disease (ESKD), and among kidney transplant (KT) candidates and recipients [2–6]. Pre-transplant frailty is associated with unfavorable outcomes after transplantation, such as delayed graft function, longer length of hospital stay, early hospital readmission, immunosuppression intolerance and higher mortality [6–10].

According to a recent meta-analysis from the US, the overall pooled prevalence of frailty is estimated at 17.1% among the transplant candidates [11]. Because of the growing geriatric population on dialysis, older candidates are accepted for transplantation to a greater extent. A study from the US showed that candidates over the age of 65 years are 1.79 times more likely to be frail compared with those under 65 years old [12]. Although frailty prevalence increases with age, a rate of 18.8% has also been reported in candidates aged between 18 and 64 years old [12]. Moreover, frail candidates appear to have a lower chance of receiving a KT and a higher risk of waitlist mortality compared with non-frail candidates [13, 14].

To date, there are limited data on knowledge about frailty and the use of tools to assess it among KT professionals. According to a US survey study on transplant centers’ perceptions of frailty assessment, although 98.4% of specialists considered frailty a valuable parameter for evaluating candidates, only 25% of centers routinely performed frailty assessments in 2020 [15].

Understanding the level of frailty knowledge among transplant physicians and their current practices is crucial for addressing the needs of vulnerable transplant candidates and recipients. This survey was designed to investigate perceptions regarding frailty and its assessment among transplant physicians in European countries, as well as the challenges that are faced in implementing frailty assessment and management in clinical practice. We anticipate that integrating knowledge on frailty into clinical workflows will contribute to a more personalized and optimized evaluation process for transplant candidates. In addition, understanding current clinical practices related to frailty assessment in European transplant centers may help raise awareness, emphasize the importance of frailty and pave the way for future guidelines.

## MATERIALS AND METHODS

### Survey design

The European Renal Association DESCaRTES working group developed an online questionnaire to assess frailty practices in kidney transplantation in Europe. The questions of the questionnaire were inspired by the US survey study investigating transplant physicians’ frailty perception in 2020 to enable comparison with the practice of European centers [15]. The survey was created using Microsoft Forms (Microsoft Corporation, Redmond, WA, USA). Survey questions were evaluated by DESCaRTES working group members before finalization.

The survey consisted of 28 branched questions, with the number depending on respondents’ answers. Questions were divided into three sections. The first section consisted of questions on the general characteristics of the transplant centers, the second section gauged the respondents’ perceptions on frailty assessments in kidney transplantation, and the third section was related to the actual assessment and management of frailty in clinical practice.

Descriptive variables encompassed physician characteristics (e.g. years of experience, dichotomized at <10 or ≥10 years based on clinical expertise thresholds), center characteristics (e.g. annual transplant volume, categorized as <50 or ≥50) and country of practice. Outcomes included frailty perceptions (e.g. binary agreement on frailty as a risk factor for adverse outcomes, measured via yes/no responses) and practices (e.g. frequency of assessment, categorized as “never,” “sometimes,” “routinely” or “in selected patients”).

Survey questions are provided in Supplementary Material.

### Survey distribution

A link to the survey, accompanied by an introductory email, was dispatched to ordinary members of the DESCaRTES working group living in European countries. Additionally, DESCaRTES working group board members contacted participants directly via their networks. The survey link was actively distributed from October 2024 to April 2025.

To ensure diversity and minimize duplication, each transplant center was allowed to submit only one survey response, and the survey had to be submitted by a transplant physician with expertise in both pre-transplant assessment and post-transplant patient care.

To mitigate selection bias, the survey targeted a broad network of DESCaRTES ordinary members across Europe, aiming for representation from at least 20 countries. Anonymous data collection was used to reduce social desirability bias in self-reported practices. Non-response bias was addressed through follow-up emails to initial non-responders, though reasons for non-participation were not systematically recorded. Measurement bias was minimized by pre-testing questions for clarity and branching logic to ensure relevant responses.

We anticipated a response rate of 30%–40% based on similar transplant surveys [15]. No formal power calculation was performed given the descriptive, exploratory nature of the study, but the sample size was deemed sufficient for estimating proportions with reasonable precision (e.g. margin of error <10% for key binary outcomes at 95% confidence).

### Data analysis

The data were collected anonymously through Microsoft Forms, an online survey tool. Descriptive statistics, including frequencies and percentages, were employed for data representation. The percentages of responses to all questions were calculated by dividing the total number of answers by the number of respondents. Chi-square tests were used to compare frailty assessment practices across center volume categories defined as less or more than 50 transplants per year. We used SPSS Statistics V22.0 (IBM, Armonk, NY, USA) for all statistical analyses.

## RESULTS

**Figure 1:**
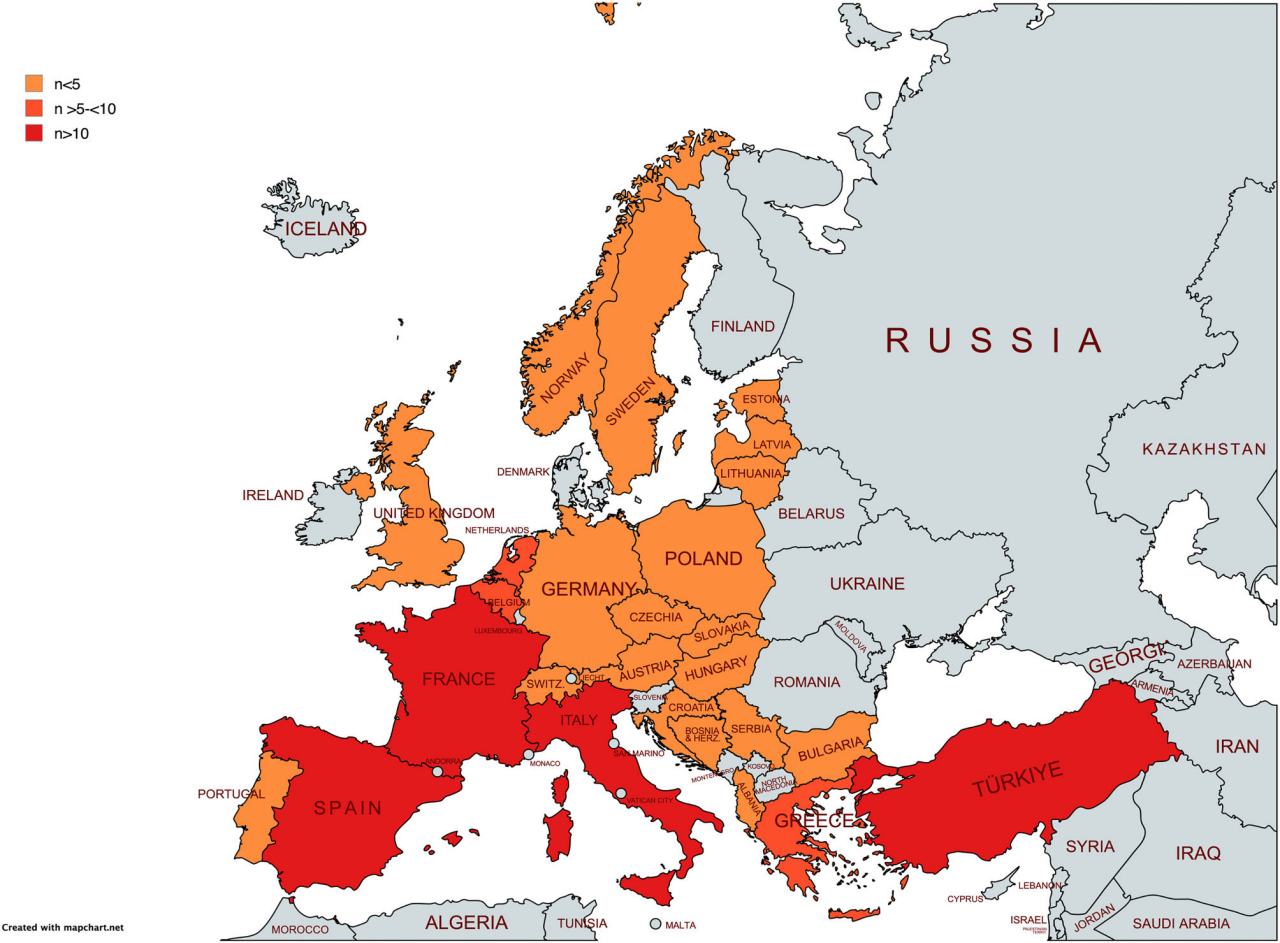
The graphical illustration of the European countries represented in the survey.

The survey was sent to 342 potential respondents, and 134 responses from 26 countries were received (Fig. 1, mapchart), yielding a response rate of approximately 39.2%.

### Participant transplant center and physician characteristics

**Figure 2:**
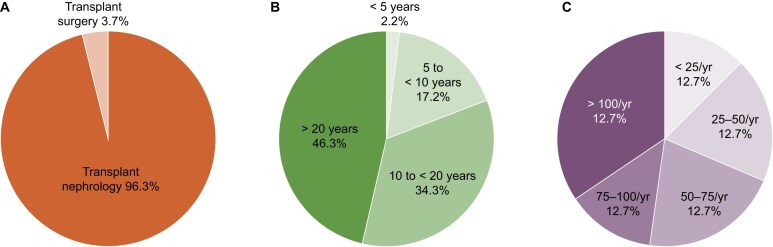
Distribution of respondent physicians’ and centers’ characteristics. (**A**) Area of practice; (**B**) experience year in clinical practice; (**C**) respondent centers’ transplant volume

The respondents comprised 129 (96.3%) nephrologists and 5 (3.7%) surgeons. Of the respondents, 80.6% had at least 10 years of experience in the transplant field, and 68.7% of the responding centers performed 50 or more transplants annually (Fig. 2). The number of centers performing primarily deceased donor transplantation (>50%) was 101 (75.3%). The proportion of preemptive kidney transplantations ranged from 0% to 70% across centers.

### Frailty perception

Responses to survey questions on frailty perception in kidney transplantation are shown in Table 1. Among the 134 respondents, 67.2% (*n* = 90) reported that there is no specific chronological age beyond which KT should not be considered. The remaining 32.8% (*n* = 44) of respondents indicated an age cut-off, with some stating 75 years (*n* = 14) and some others 85 years (*n* = 17) as upper age limits.

**Table 1:** Responses to survey questions on frailty perception in kidney transplantation.

General frailty perception		All participants^a^ (*n* = 134)
1. Do you think there is a chronological age-limit when kidney transplantation should not be considered?	Yes	44 (32.8)
	No	90 (67.2)
If yes:		
1.1 What is the age-limit at which transplantation should not be considered?	Above 75 years of age	14 (10.4)
	Above 80 years of age	8 (6.0)
	Above 85 years of age	17 (12.7)
	Other	5 (3.7)
2. Do you think frailty can be reversed in ESKD patients?	Yes	65 (48.5)
	No	17 (12.7)
	I am unsure	52 (38.8)
If yes:		
2.1 Please rank the following interventions in order of their usefulness for improving frailty (1st to 5th indicating which is most and least useful)	Physical exercise and rehabilitation programs	28 (20.9)
	Optimization of dialysis treatment	7 (5.2)
	Nutritional counseling and dietary supplements	6 (4.5)
	Psychosocial support and counseling	2 (1.5)
	Kidney transplantation	22 (16.4)
3. Do you believe frailty is a risk factor for adverse outcomes during waiting time in patients on the KT waiting list?	Yes	132 (98.5)
	No	1 (0.7)
	I am unsure	1 (0.7)
4. Do you believe a standardized frailty assessment is an important part of the evaluation process of potential KT candidates?	Strongly disagree	7 (5.2)
	Disagree	9 (6.7)
	Neutral	5 (3.7)
	Agree	64 (47.8)
	Strongly agree	49 (36.6)
5. Do you believe that pre-transplant frailty is a risk factor for adverse outcomes after transplantation?	Yes	131 (97.8)
	No	1 (0.7)
	I am unsure	2 (1.5)
6. Do you believe that a standardized frailty assessment is a useful method in KT recipient follow-up after transplantation?	Yes	100 (74.6)
	No	8 (6.0)
	I am unsure	26 (19.4)

a Percentages are described in parentheses.

Frailty was overwhelmingly recognized as a risk factor for adverse outcomes both before (98.5%) and after (97.8%) KT. Notably, opinions were split on whether frailty in younger patients is less concerning than in older adults; half of the respondents disagreed with this notion, while 41% believed it to be true and 9% were unsure.

Among the participants, 48.5% (*n* = 65) believed that frailty can be reversed in ESKD patients. In contrast, 12.7% (*n* = 17) did not believe that it can be reversed, while 38.8% (*n* = 52) were uncertain.

Among those who believed frailty can be reversed, physical exercise and rehabilitation programs were the most frequently reported interventions for improving frailty (*n* = 28, 20.9%), followed by KT (*n* = 22, 16.4%). Psychosocial support and counseling was the least frequently selected option for improving frailty in ESKD patients (*n* = 2, 1.5%). Furthermore, 57.5% (*n* = 77) believed that frailty is likely to improve after transplantation.

A majority of respondents (84.4%) agreed that frailty should be an important part of the KT eligibility evaluation. Only 11.2% (*n* = 15) believed that adequate training and educational resources for frailty assessment are currently available for transplant physicians. While 52.2% felt resources were somewhat adequate, 36.6% stated that such tools were insufficient.

### Frailty practice

#### Pre-transplant

Table 2 summarizes the current practices of frailty assessment in the evaluation of KT candidates. Frailty is not routinely assessed in most of the centers: 29.9% of respondents reported never evaluating frailty during KT eligibility assessments, while 38.8% did so occasionally. Only 6.7% assessed frailty routinely, and 24.6% evaluated frailty only in patients meeting predefined criteria, most commonly, based on age and/or comorbidities. Pre-existing cardiovascular disease, diabetes and prolonged dialysis duration were the most common clinical conditions prompting to consider assessment of frailty. Among these, dialysis duration showed no clear threshold, with 5- and 10-year durations being equally considered as a risk factor for frailty. Low body mass index (BMI) (<18 kg/m^2^) was stated more frequently than obesity as a frailty concern.

**Table 2:** Current practices of frailty assessment among transplant physicians evaluating KT candidates.

Practices of frailty in KT candidates		All participants^a^ (*n* = 134)
1. Do you currently perform a standardized frailty assessment as part of the evaluation process for KT eligibility in your clinical practice?	Yes, always	9 (6.7)
	Yes, systematically in a predefined subgroup of patients	33 (24.6)
	Sometimes (no predefined criteria)	52 (38.8)
	No, never	40 (29.9)
If yes:		
1.1. In which patients do you perform a frailty assessment?	All candidates above a certain age cut-off	20 (14.9)
	Specific predefined criteria based on comorbidities (± age)	22 (16.4)
	On a case-by-case basis	43 (32.0)
1.1.1. At what age do you perform a frailty assessment?	In patients above 60 years	1 (0.7)
	In patients above 65 years	4 (3.0)
	In patients above 70 years	9 (6.7)
	In patients above 75 years	5 (3.7)
	Other	1 (0.7)
1.1.2. What comorbidities do you take into consideration when identifying individuals for frailty assessments? (Select all that apply)	Cardiovascular disease (ischemic heart disease, cerebrovascular disease, peripheral arterial vascular disease)	85 (63.4)
	Diabetes mellitus	59 (44.0)
	Longer dialysis vintage (please indicate year cutoff)	52 (38.8)
	BMI >30	32 (23.9)
	BMI <18	43 (32.1)
	Other	7 (5.2)
1.2. When evaluating frailty, please select up to five of the following variables that you consider most important	Physical activity	78 (58.2)
	Grip strength	29 (21.6)
	Gait speed	18 (13.4)
	Sit-to-stand ability	36 (26.9)
	Skeletal muscle mass	39 (29.1)
	Unintentional weight loss	36 (26.9)
	Cognitive function	78 (58.2)
	Depression or anxiety	22 (16.4)
	Laboratory markers	12 (9.0)
	Need for assistance with activities of daily living	57 (42.5)
	Comorbidities (e.g. diabetes mellitus, cardiovascular diseases)	43 (32.1)
	Other (Charlson comorbidity index)	1 (0.7)
1.3. What tool or assessment method do you routinely use for assessing frailty? (Select all that apply)	Fried Frailty Phenotype	21 (15.7)
	Frailty Index	35 (26.1)
	FRAIL scale	23 (17.2)
	Functional status assessments (e.g. KDQOL, SF-36 or PCS)	23 (17.2)
	Stair-climbing assessment	9 (6.7)
	Timed walk test	17 (12.7)
	Other	16 (11.9)
1.4. Please indicate who conducts these assessments (select all that apply)	Nurse	37 (27.6)
	Transplant physician	56 (41.8)
	Nutritionist	9 (6.7)
	Physical therapist	3 (2.2)
	Transplant coordinator	8 (6.0)
	Geriatrician	27 (20.1)
	Other	4 (3.0)
1.5. How do you utilize the results of a standardized frailty assessment? (Select all that apply)	Decision-making regarding listing	58 (43.2)
	Decision-making regarding deceased kidney transplantation	24 (17.9)
	Decision making regarding living kidney transplantation	32 (23.9)
	Decision on the choice of induction therapy	23 (17.2)
	Decision to tailor immunosuppression	37 (27.6)
	Decision about rehabilitation prior transplantation	38 (28.4)
	All of the above	24 (17.9)
	Other	7 (5.2)
2. If you do not assess frailty as part of the evaluation process before transplantation, what is the most important barrier to implementing standardized frailty assessments in your practice? (Select all that apply)	Lack of time for conducting assessments	19 (14.2)
	Limited access to frailty assessment tools	15 (11.2)
	Insufficient trained or experienced staff for the assessments	31 (23.1)
	Resistance from patients to participate in assessments	2 (1.5)
	Lack of standardized guidelines for frailty assessment in KT candidates	20 (14.9)
	Uncertainty about how to incorporate frailty assessment results into decision-making	19 (14.2)
	Other	0 (0.0)
3. What improvements or resources would help you incorporate frailty assessments effectively in your practice? (select all that apply)	Comprehensive training programs on frailty assessment	77 (57.5)
	Access to user-friendly frailty assessment tools	94 (70.1)
	Clear guideline on how to interpret frailty assessment results	90 (67.2)
	Collaboration with specialists in geriatric medicine	60 (44.8)
	Increase support staff to assist with assessments	63 (47.0)
	Integration of frailty assessment into electronic health records	55 (41.0)
	Other	4 (3.0)

a Percentages are described in parentheses. KDQOL, Kidney Disease Quality of Life; PCS, Physical Component Score.

When evaluating frailty, physical activity (*n* = 78, 58.2%), cognitive function (*n* = 78, 58.2%) and dependence in activities of daily living (*n* = 57, 42.5%) were the most considered domains. The most frequently used assessment tool was the Frailty Index (FI), which was reported by 26.1% of programs (*n* = 35). Other tools included the Fried Frailty Phenotype (FFP) (*n* = 21, 15.7%), the FRAIL scale (FS) (*n* = 23, 17.2%), functional status assessments such as the Short Form (SF)-36 (*n* = 23, 17.2%) and time-intensive tests [6-min walking test (*n* = 17, 12.7%) or stair climbing time (*n* = 9, 6.7%)]. Some centers reported using alternate or locally adapted tools, likely based on experience and available resources.

Frailty assessments were most often conducted by transplant physicians (*n* = 56, 41.8%), followed by nurses (*n* = 37, 27.6%) and geriatricians (*n* = 27, 20.1%). The results of frailty assessments were mainly utilized for decisions on listing (*n* = 58, 43.2%), as well as to guide pre-transplant rehabilitation planning (*n* = 38, 28.4%).

When asked about barriers to implementing frailty assessments, respondents most frequently chose the option of insufficient trained or experienced staff for the assessments (*n* = 31, 23.1%), followed by the lack of standardized guidelines (*n* = 20, 14.9%). Time constraints (*n* = 19, 14.2%), limited access to assessment tools (*n* = 15, 11.2%) and uncertainty regarding how to apply frailty findings in clinical decision-making (*n* = 19, 14.2%) were also noted as obstacles. Participants emphasized that user-friendly tools (*n* = 94, 70.1%), clear practice guidelines (*n* = 90, 67.2%) and comprehensive training programs (*n* = 77, 57.5%) would facilitate broader adoption of frailty assessments.

In the analyses comparing respondents’ characteristics, physicians from relatively high-volume centers (>50 transplants/year) were more likely to report performing frailty assessment always (7.4% vs 4.8%) and according to predefined criteria (30.9% vs 9.5%) compared with those from lower-volume centers (*P* = .048). No significant differences were observed in the current practice of frailty assessment during transplant evaluation when comparing respondents’ years of clinical experience.

#### Post-transplant

Most respondents (74.6%, *n* = 100) agreed that standardized frailty assessments are valuable in post-transplant follow-up, though only one-third (33.6%) reported using them on a case-by-case basis, and nearly half (49.3%) did not assess frailty routinely after transplantation. The most widely recognized benefit of post-transplant frailty assessment was the ability to identify patients at higher risk for complications (*n* = 113, 84.3%) (Table 3).

**Table 3:** Frailty assessment practices in KT recipients.

Frailty in KT recipients		All participants^a^ (*n* = 134)
1. Do you perform a standardized frailty assessment after transplantation as a part of follow-up?	Yes, in all recipients	2 (1.5)
	Yes, in all recipients above a certain age cutoff (please indicate age cutoff)	3 (2.2)
	Yes, according to specific predefined criteria based on comorbidities (± age)	9 (6.7)
	Yes, but only in pre-transplant frail recipients	8 (6.0)
	On a case-by-case basis	45 (33.6)
	No	66 (49.3)
2. In your opinion, what are the primary benefits of conducting frailty assessments in KT recipients? (Select all that apply)	Identifying patients at higher risk for post-transplant complications	113 (84.3)
	Tailoring post-transplant immunosuppression to individual patient needs	71 (53.0)
	Improving overall transplant outcomes	85 (63.4)
	Other	7 (5.2)

a Percentages are described in parentheses.

## DISCUSSION

Our findings highlight that frailty is widely accepted as a significant risk factor for inferior outcomes in KT candidates and recipients. Most respondents endorsed its integration into both pre- and post-transplant evaluations. Despite this, routine standardized assessment remains limited, reflecting a significant gap between knowledge and actual practice.

Although an increasing number of studies have examined the impact of frailty on KT outcomes in recent years [2, 6, 7, 11, 14, 16], there is insufficient information on how clinicians perceive frailty in transplant candidates and recipients and how they use it in practice. According to our survey, approximately one-third of respondents do not assess frailty. Similarly, a study conducted in US transplant centers showed that 31% of the respondents had never assessed frailty at the time of evaluation. Forty-four percent of them assessed frailty sometimes, and 25% assessed it routinely [15]. In our survey, only 6.7% of the respondents assessed frailty routinely. Even though routine frailty assessment was less frequently reported in European transplant centers, the overall use of frailty assessment, whether performed routinely, occasionally or in selected patient groups, was comparable to that reported in US survey. This suggests that the observed differences may relate less to clinical awareness and more to structural and organizational factors, including differences in regulatory frameworks or allocation policies. Additionally, it should be noted that patients’ demographics may be different from those in European centers. In 24% of the transplant centers, frailty was assessed only in a specific subgroup of patients. The candidate’s age was a common consideration. Given that the KDIGO Clinical Practice Guideline on the Evaluation and Management of Candidates for Kidney Transplantation recommends that patients with ESKD should not be excluded from KT based on age alone [17], the growing geriatric population, defined as individuals older than 65 years, has drawn attention to the potential importance of frailty assessment in transplant practice. Nevertheless, focusing solely on older age may overlook the broader relevance of frailty because frailty can affect individuals of all ages, including younger patients with chronic diseases such as chronic kidney disease [12, 18]. Indeed, the KDIGO guideline for candidates recommends that patients should not be excluded from transplantation based solely on age; instead, candidates should be assessed in the context of comorbidities, including frailty [17].

Moreover, our study indicated that irrespective of age, patients with cardiovascular disease, diabetes, longer dialysis duration, and a BMI <18 kg/m² were more likely to be identified by respondents as frail during evaluation for KT. These findings align with previous studies highlighting that frailty is not solely age-dependent but is strongly associated with clinical factors in patients being assessed for transplantation [14, 19–21]. In particular, Perez Fernandez *et al*. showed that frailty in KT candidates was more prevalent among those with diabetes and cardiovascular disease, all of which impair physiologic reserve and functional capacity [14]. Similarly, Johansen *et al*. found that frailty was more common in waitlisted patients with comorbidities and lower serum albumin, further supporting the clinical impression that poor nutritional status and sarcopenia, often reflected in low BMI, are important indicators of vulnerability [19]. Altogether, these studies reinforce the idea that frailty among transplant candidates is shaped by the accumulation of comorbid conditions and the physiological impact of dialysis, rather than age alone. This may explain why transplant physicians were more likely to consider such patients as frail during pre-transplant evaluation.

A wide range of frailty assessment tools were reported to be in use, with no single method universally adopted. Although more than 70 frailty tools are described in the literature, they vary in complexity, time requirements and the specific domains assessed [22]. Assessment of frailty ideally includes tools that evaluate physical, cognitive and psychosocial domains to capture the full scope of a patient’s functional status [23]. Among our respondents, physical function emerged as the most critical domain, supporting the notion that some degree of physical dysfunction is an inevitable consequence of ageing and/or chronic disease and represents a key feature of frailty in many adults [24].

While the FFP is a widely validated tool, its clinical application may be constrained by the need for specific equipment to measure handgrip strength and gait speed [1]. FFP, FI, FS and timed walk tests/stair-climb assessments are the more commonly used tools reported in our survey. The FI and FS are user-friendly, time-efficient, include morbidity/disability status and do not require additional measurements. However, their self-reported components may limit their use [25]. Comparing our findings with a 2020 US survey study, it appears that timed walk tests and stair-climb assessments, once popular, are now less favored due to limited availability of suitable spaces for sustainable implementation and the perception that these tests do not adequately capture other domains of frailty other than physical status [1^5^, ^25^].

There are limited studies comparing different frailty assessment tools in transplant candidates. Pérez-Sáez *et al*. examined the agreement between the Physical Frailty Phenotype and the FS in 451 KT candidates [26]. Their findings showed poor agreement between the two tools, with the FS potentially misclassifying frail or pre-frail patients in better health as robust. Observational and comparative longitudinal studies are needed to identify the most appropriate frailty assessment tool for use in kidney transplantation practice.

Several barriers hinder the widespread adoption of frailty assessments in transplant programs. We observed that transplant physicians are the primary assessors, which may be related to familiar challenges such as lack of time or absence of sufficiently trained staff other than transplant physicians. Geriatricians were involved in frailty assessments in only 20% of participating centers. Strengthening collaboration with geriatricians in transplant practice is essential, as they are the specialists most experienced and knowledgeable in the concept of frailty. Additionally, the absence of clear guidelines is another major obstacle. In line with these findings, we can conclude that accessible, easy-to-implement assessment methods and introducing a consensus on the best assessment approach should be prioritized. Then, training programs should be expanded in order to generalize the frailty assessments in transplant practice. Interestingly, participants from high-volume centers were more likely to perform frailty testing compared with those from low-volume centers. This tendency may be attributed to several advantages of such centers, including larger staff capacity, superior resource infrastructure, greater involvement in research and a higher admission rate of patients with older age and/or comorbidities.

Approximately half of the participants believed that frailty can be reversed, and our survey indicates that physical exercise and rehabilitation programs were deemed beneficial interventions for reversing or improving frailty. Several modest-sized clinical trials examining prehabilitation in KT candidates have produced encouraging results, including significant improvements in frailty components, such as muscular strength, physical function and regular physical activity levels [27–29]. Prehabilitation studies also indicate potential for significant cost-effectiveness by decreasing post-operative hospital stay duration [30].

Recently, the European Society for Organ Transplantation has agreed that prehabilitation is possible, acceptable and safe [31]. A randomized study investigating an 8-week, exercise-based prehabilitation program in KT candidates demonstrated that prehabilitation, compared with standard care, significantly improved exercise capacity, muscle strength and muscle size, particularly in frail candidates, highlighting the potential benefits of structured prehabilitation prior to transplantation [32]. However, our survey indicates that despite broad recognition of its value, further efforts are needed to overcome barriers to implementing prehabilitation in the transplant community. Larger prospective trials to evaluate that are on the way [33].

Furthermore, kidney transplantation itself has also been considered as an intervention for improving frailty [34–37]. Aroca-Martinez *et al*. [34] assessed frailty using the Clinical Frailty Scale (CFS) in 57 KT recipients, reporting a significant improvement in frailty scores, with the median CFS decreasing from 4 (vulnerable) to 3 (robust), 6 months post-transplant (*P* < .01). Dos Santos Mantovani *et al*. [35] observed a 69.9% reduction in the prevalence of frailty measured by physical frailty phenotype criteria at 12 months following kidney transplantation. In contrast, Quint *et al*. reported an increase in frailty prevalence from 17% to 26.7% after transplantation with a mean follow-up of 22.8 ± 8.3 months [38]. Restoring kidney function and subsequent improvements in micronutrient deficiencies, physical activity, sarcopenia and even chronic inflammation following transplantation may all contribute to the reversal of frailty. However, it should be noted that KT is a highly dynamic process that may result in a transition from frail to non-frail in some recipients, while others may transition from a non-frail to a frail state. In our survey study, the high level of uncertainty regarding frailty reversibility likely reflects the current state of evidence rather than a lack of awareness among clinicians.

### Limitations

There are several limitations to consider when interpreting the results of this survey. First, the absence of an explicit, prespecified definition of “standardized frailty assessment” may have introduced variability in interpretation across participants. Second, although participating countries cover almost all European border countries (*n* = 26), the absence of country-specific response rates limits the generalizability of our findings to each country and precludes a robust assessment of potential regional differences. Third, there is a possibility that respondents reported their “ideal” behavior rather than their routine clinical behavior. This tendency toward socially desirable responses is common in self-reported surveys and might have influenced the accuracy of the reported data. Similarly, the survey may not distinguish between individual and institutional (center-level) approaches in the follow-up care of patients. Lastly, centers with a pre-existing interest in frailty may have been more inclined to participate, potentially introducing selection bias and further skewing the results. However, the survey was distributed broadly to all DESCaRTES working group ordinary members across Europe, without targeting centers known to have a specific interest in frailty, thereby reducing preferential sampling. Also, follow-up emails were employed to initial non-responders to encourage participation and reduce non-response bias. Additionally, perspectives from geriatricians and pediatric specialists were not reflected in this study. Routine implementation of frailty assessments in kidney transplantation cannot be effectively achieved without close collaboration with geriatricians. Similarly, input from pediatric specialists is essential, as frailty in children and adolescents represents a distinct construct driven by disease-related vulnerability rather than age-related physiological decline.

In conclusion, transplant physicians recognize the importance of frailty but do not consistently apply this knowledge in their clinical practice. Implementing standardized guidelines specific to transplantation may help promote routine frailty screening and formal assessments. In addition, further research is needed to develop strategies to promote best practices for the pre- and rehabilitation of frail KT candidates and recipients.

## LIST OF CONTRIBUTORS

Respondents were invited to provide their credentials at the conclusion of the survey to be acknowledged as contributors. Those who opted not to share their credentials are consequently excluded from this list. Only one response per center was included in the final analysis.

Nada Kanaan, Cliniques Universitaires Saint-Luc, Brussels, Belgium; Dirk Kuypers, University Hospitals, Leuven, Belgium; Andrea Ranghino, Polytechnic University of Marche, Nephrology, Dialysis and Kidney Transplant Unit, AOU delle Marche Hospital, Ancona, Italy; Silvie Rajnochova Bloudickova, Department of Nephrology, Transplant Center, Institute for Clinical and Experimental Medicine, Videnska, Prague, Czech Republic; Matej Vnučák, Transplant-Nephrology Department, University Hospital Martin, Martin, Slovakia; Jan Burkert, Transplant Center, University Hospital Motol, Prague, Czech Republic; Dilan Dabare, Department of Renal Transplantation, University Hospitals Birmingham, Birmingham, UK; Huseyin Kocak, Akdeniz University Transplantation Center, Antalya, Türkiye; Berna Yelken, Basaksehir Cam and Sakura City Hospital, Istanbul, Türkiye; Hamad Dheir, Sakarya University Faculty of Medicine, Division of Nephrology, Sakarya, Türkiye; Dilek Barutcu Atas, Marmara University School of Medicine, Department of Internal Medicine, Division of Nephrology, Istanbul, Türkiye; Ana M. Ramos, Fundación Jiménez Díaz, Madrid, Spain; Alex Gutierrez-Dalmau, Hospital Universitario Miguel Servet, Aragón Health Research Institute (IIS Aragón), Zaragoza, Spain; Isabel Beneyto Castelló, Hospital La Fe, Valencia, Spain; Ana Vila, Hospital Germans Trias i Pujol, Badalona, Spain; Umberto Maggiore, Department of Medicine and Surgery, Parma, Italy; Auxiliadora Mazuecos, Hospital Puerta del Mar, Cadiz, Spain; Cristina Jorge, ULS de São José, Lisbon, Portugal; Kadir Gokhan Atilgan, Ankara Etlik City Hospital, Ankara, Türkiye; María José Pérez Sáez, Hospital del Mar, Barcelona, Spain; Gulay Asci, Ege University School of Medicine, Izmir, Türkiye; Gizem Kumru, Ankara University Faculty of Medicine, Department of Nephrology, Ankara, Türkiye; Michaela Matyskova Kubisova, Faculty Hospital, Hradec Kralove, Czech Republic; Gaetano La Manna, Nephrology, Dialysis and Kidney Transplant Unit, IRCCS Azienda Ospedaliero Universitaria di Bologna, Department of Medical and Surgical Sciences (DIMEC), Alma Mater Studiorum University of Bologna, Bologna, Italy; Marielle Gelens, Maastricht University Medical Centre, Maastricht, Netherlands; Enrico Eugenio Minetti, Nephrology Unit, Grande Ospedale Metropolitano Niguarda, Milan, Italy; Aae De Joode, University of Groningen, University Medical Center Groningen, Department of Internal Medicine, Division of Nephrology, Groningen, The Netherlands; Antoine Bouquegneau, Division of Nephrology-Dialysis and Transplantation, University of Liège (Uliège), CHU Sart Tilman, Liège, Belgium; H. Asuman Yavuz, Medicalpark Hospital, Antalya, Türkiye; Fatih Mehmet Erdur, Gaziantep University, Faculty of Medicine, Department of Nephrology, Gaziantep, Türkiye; Kathrin Eller, Medical University of Graz, Division of Nephrology, Graz, Austria; Sultan Ozkurt, Osmangazi University Faculty of Medicine, Department of Nephrology, Eskisehir, Türkiye; Bruno Watschinger, Medical University of Vienna, Vienna, Austria; Paloma L Martin-Moreno, Nephrology Department, Clínica Universidad de Navarra, IDISNA, Navarra Institute for Health Research, Pamplona, Spain; Giacomo Mori, Division of Nephrology, Dialysis and Kidney Transplantation, University Hospital of Modena, Modena, Italy; Marco Vivarelli, Polytechnic University of Marche, Ospedali Riuniti delle Marche, Ancona, Italy; Zuzana Zilinska, University Hospital, Bratislava, Slovakia; Concetta Catalano, Nephrology, Dialysis and Kidney Transplantation Unit, Hôpital Erasme, Hôpital Universitaire de Bruxelles, Brussels, Belgium; Martina Koch, University Medical Center Mainz, Mainz, Germany; Martina Guthoff, University of Tübingen, Tübingen, Germany; Meral Mese, Kartal Dr Lütfi Kırdar City Hospital, Istanbul, Türkiye; Licia Peruzzi, Regina Margherita Hospital, Turin, Italy; Anne-Élisabeth Heng, CHU Clermont-Ferrand, Clermont-Ferrand, France; Maria Magott, University Hospital Wrocław, Wrocław, Poland; Marta Crespo, Hospital del Mar, Barcelona, Spain; Izabela Zakrocka, Medical University of Lublin, Lublin, Poland; Safak Mirioglu, Bezmialem Vakıf University Hospital, Istanbul, Türkiye; Milena Nikolova, Medical University of Sofia, Sofia, Bulgaria; Eva Gavela, Nephrology Department, Hospital Dr Peset, Valencia, Spain; Alessandra Marega, Nephrology, Dialysis and Kidney Transplant, ASUFC Udine, Udine, Italy; Chrysanthi Skalioti, Assistant Professor of Nephrology and Kidney Transplantation, Department of Nephrology and Kidney Transplantation, School of Medicine, National and Kapodistrian University of Athens, Laiko Hospital, Athens, Greece; Enisa Mesic, UKC Tuzla, Tuzla, Bosnia and Herzegovina; Mehdi Maanaoui, CHU Lille, University of Lille, Lille, France; Ismail Baloglu, Necmettin Erbakan University, Konya, Türkiye; Alexandre Hertig, Foch Hospital, Suresnes, France; Vincenzo Cantaluppi, University of Piemonte Orientale (UPO), AOU Maggiore della Carità, Novara, Italy; Przemyslaw Rutkowski, Medical University of Gdańsk, Gdańsk, Poland; Maria Pippias, North Bristol NHS Trust, Bristol, UK; Olta Dhamo, Albanian-Turkish Friendship Hospital, Fier, Albania; Maria Nikodimopoulou, Transplant Unit, Aristotle University, Hippokration Hospital, Thessaloniki, Greece; Dela Golshayan, Lausanne University Hospital, Lausanne, Switzerland; Bojana Šimunov, University Hospital Merkur, Zagreb, Croatia; Emilio Rodrigo, Nephrology Service, University Hospital Marqués de Valdecilla/IDIVAL, Santander, Spain; Andrea Buscaroli, University of Bologna, Bologna, Italy; Mai Rosenberg, MD, PhD, Tartu University Hospital, Tartu, Estonia; Eleni Theodoropoulou, Alexandra General Hospital, Athens, Greece; Rita Leal, Nephrology Department, ULS Coimbra, Coimbra, Portugal; Diana Rodríguez-Espinosa, Hospital Clínic Barcelona, Barcelona, Spain; Simon Ville, Institut de Transplantation Urologie Néphrologie (ITUN), CHU Nantes, Nantes, France; Nereida Spahia, University Hospital Center “Nënë Tereza”, Tirana, Albania; Valeria Corradetti, IRCCS Azienda Ospedaliero Universitaria di Bologna, Bologna, Italy; Külli Kõlvald, Tartu University Hospital, Tartu, Estonia; Dany Anglicheau, Necker Hospital, Paris, France; Evangelia Ntounousi, Nephrology Department, Faculty of Medicine, University of Ioannina, Ioannina, Greece; Peggy Perrin, Hôpitaux Universitaires de Strasbourg, Strasbourg, France; J. Tourret, Sorbonne-Université, Assistance Publique–Hôpitaux de Paris (AP-HP), Paris, France; Sunil Daga, Leeds Teaching Hospitals NHS Trust, Leeds, UK; Ruth Fergie, Regional Nephrology and Transplant Unit, Belfast City Hospital; Centre for Public Health, Queen’s University Belfast, Belfast, UK.

## Supplementary Material

sfag065_Supplemental_File

## Data Availability

The article’s data will be shared with the corresponding author at a reasonable request.
