# Supplementary material for: Frailty assessment in kidney transplantation: insights from a European survey
Source: Clin Kidney J. 2026 Feb 26;19(5):sfag065. doi: 10.1093/ckj/sfag065 (PMC13133629; doi:10.1093/ckj/sfag065)
Supplement: sfag065_Supplemental_File [file sfag065_supplemental_file.docx]

Supplemental S1– the full survey

**Introduction**

As a European transplant nephrologist, your expertise and experience in the field are invaluable. We are conducting a survey to gain insight into current practice patterns of frailty assessments pre- and post-transplant patient care in European transplant centers, supported by the ERA Descartes Working Group. Your participation in this survey is crucial to help us better understand current practices and identify areas for improvement.

The survey is very easy, and will take approximately 5-10 minutes to complete.  It can be accessed through this link:

Please note that we allow only 1 respondent per transplant center. If you prefer, you can also forward the survey to a colleague (but note that we are specifically looking for physicians active in pre-transplant evaluation, as well as post-transplant patient care)

All responses will be kept strictly confidential and will only be used for research purposes.

At the end of the survey, you will be invited to leave your credentials, so that we can acknowledge you as a collaborator in the publication that may result from the survey.

Your participation in this study is greatly appreciated, and we hope that you will take the time to complete the survey. If you have any questions or concerns, please do not hesitate to contact us.

Thank you for your time and participation

***Type of practice***

**Please select your country of practice**

What is your area of practice?

1. Transplant nephrology
2. Transplant surgery
3. Other, please specify

**How many years of experience do you have in your practice?**

1. <5 years
2. 5-<10 years
3. 10-<20years
4. >20 years

**Please describe the institution where you work:**

1. University hospital
2. Public hospital
3. Private hospital
4. Other, please specify

**What is the average number of kidney transplantations your center performs annually?**

1. < 25 /yr
2. 25-50 /yr
3. 50-75 /yr
4. 75-100 /yr
5. > 100 /yr

**What is the percentage of living donor kidney transplantations in your center's overall transplant activity?**

**What is the percentage of preemptive kidney transplantations in your center's overall transplant activity?**

***General Questions***

1. **Do you think there is a chronological age-limit when kidney transplantation should not be considered?**

Yes

No

1. **If yes, what is the age-limit at which transplantation should not be considered?**

above 75 years of age

above 80 years of age

above 85 years of age

other

1. **How familiar are you with the concept of frailty assessment?**

Very familiar

Somewhat familiar

Not familiar at all

1. **Do you believe a standardized frailty assessment is an important part of the evaluation process of potential kidney transplant candidates?**

Strongly disagree

Disagree

Neutral

Agree

Strongly agree

1. **Do you believe frailty is a risk factor for adverse outcomes during waiting time in patients on the kidney transplant waiting list?**

Yes

No

I am unsure

1. **Do you believe that pre-transplant frailty is a risk factor for adverse outcomes after transplantation?**

Yes

No

I am unsure

1. **Do you think frailty can be reversed in end-stage kidney disease patients?**

Yes

No

I am unsure

1. **If you answered "yes" to question 5, please rank the following interventions in order of their usefulness for improving frailty (Drag the lines from 1st to 5th indicating which is most and least useful)**

Physical exercise and rehabilitation programs

Optimization of dialysis treatment

Nutritional counseling and dietary supplements

Psychosocial support and counseling

Kidney Transplantation

1. **Do you believe frailty is likely to improve after kidney transplantation?**

Yes

No

I am unsure

1. **Do you believe that frailty in younger people is less concerning than frailty in the elderly?**

Yes

No

I am unsure

1. **Do you think there are adequate training and education tools on frailty assessment available for transplant physicians?**

Not at all

Somewhat

Sufficiently

Very much

***Questions about Frailty in Kidney Transplant Candidates***

1. **Do you currently perform a standardized frailty assessment as part of the evaluation process for kidney transplant eligibility in your clinical practice? (If your answer is “No, never”, please skip to the question 21)**

Yes, always

Yes, systematically in a predefined subgroup of patients

Sometimes (no predefined criteria)

No, never

1. **If you currently perform standardized frailty assessments at your transplant evaluation center, please indicate who conducts these assessments (select all that apply):**

Nurse

Transplant physician

Nutritionist

Physical therapist

Transplant coordinator

Geriatrician

Other (please specify)

1. **When evaluating frailty, please select up to five of the following variables that you consider most important:**

Physical activity

Grip strength

Gait speed

Sit-to-stand ability

Skeletal muscle mass

Unintentional weight loss

Cognitive function

Depression or anxiety

Laboratory markers

Need for assistance with activities of daily living (ADL)

Comorbidities (e.g., diabetes mellitus, cardiovascular diseases)

Other (please specify)

1. **What tool or assessment method do you routinely use for assessing frailty? Select all that apply)**

Fried Frailty Phenotype

Frailty Index

FRAIL Scale

Functional status assessments (e.g., KDQOL, SF-36, or PCS)

Status of sarcopenia

Stair Climbing Assessment

Timed walk test

Other (please specify)

1. **In which patients do you perform a frailty assessment?**

All candidates

All candidates above a certain age cut-off

Specific predefined criteria based on co-morbidities (+/- age)

On a case-by-case basis

Other (please explain)

1. **At what age do you perform a frailty assessment?**

in patients above 60 years

in patients above 65 years

in patients above 70 years

in patients above 75 years

other

1. **What comorbidities do you take into consideration when identifying individuals for frailty assessments? (Select all that apply)**

Cardiovascular disease (ischemic heart disease, cerebrovascular disease, peripheral arterial vascular disease)

Diabetes mellitus

Longer dialysis vintage (please indicate year cutoff)

BMI > 30

BMI < 18

Other (please specify)

1. **How do you utilize the results of a standardized frailty assessment? (Select all that apply)**

Decision-making regarding listing

Decision-making regarding deceased kidney transplantation

Decision making regarding living kidney transplantation

Decision on the choice of induction therapy

Decision to tailor immunosuppression

Decision about rehabilitation prior transplantation

All of the above

Other (please specify)

1. **If you currently perform standardized frailty assessments in your center, how confident are you in your ability to interpret frailty assessment results?**

Not confident at all

Somewhat confident

Very confident

1. **If you do not assess frailty as part of the evaluation process before transplantation, what is the most important barrier to implementing standardized frailty assessments in your practice? (select all that apply)**

Lack of time for conducting assessments

Limited access to frailty assessment tools

Insufficient trained or experienced staff for the assessments

Resistance from patients to participate in assessments

Lack of standardized guidelines for frailty assessment in kidney transplant candidates

Uncertainty about how to incorporate frailty assessment results into decision-making

Other (please specify)

1. **If you do not currently assess frailty in your center, are you interested in implementing frailty assessments for your kidney transplant candidates?**

Yes

No

I am unsure

1. **What improvements or resources would help you incorporate frailty assessments effectively in your practice? (select all that apply)**

Comprehensive training programs on frailty assessment

Access to user-friendly frailty assessment tools

Clear guideline on how to interpret frailty assessment results

Collaboration with specialists in geriatric medicine

Increase support staff to assist with assessments

Integration of frailty assessment into electronic health records

Other (please specify)

***Questions about Frailty after Kidney Transplantation***

1. **Do you believe that a standardized frailty assessment is a useful method in kidney transplant recipient follow-up after transplantation?**

Yes

No

I am unsure

1. **Do you perform a standardized frailty assessment after transplantation as a part of follow-up?**

Yes, in all recipients

Yes, in all recipients above a certain age cut-off (please indicate age cutoff)

Yes, according to specific predefined criteria based on co-morbidities (+/- age)

Yes, but only in pre-transplant frail recipients

On a case-by-case basis

No

Other (please specify)

1. **In your opinion, what are the primary benefits of conducting frailty assessments in kidney transplant recipients? (Select all that apply)**

Identifying patients at higher risk for post-transplant complications

Tailoring post-transplant immunosuppression to individual patient needs

Improving overall transplant outcomes

Other (Please specify)

1. **If you do not currently assess frailty, are you interested in implementing standardized frailty assessments after kidney transplantation?**

Yes

No

I am unsure

1. **Do you have any additional remarks or comments regarding frailty assessments or interventions for kidney transplant candidates/recipients? (Open field)**
